# Plasma Globotriaosylsphingosine and α-Galactosidase A Activity as a Combined Screening Biomarker for Fabry Disease in a Large Japanese Cohort

**DOI:** 10.3390/cimb43010032

**Published:** 2021-06-19

**Authors:** Hiroki Maruyama, Atsumi Taguchi, Mariko Mikame, Atsushi Izawa, Naoki Morito, Kazufumi Izaki, Toshiyuki Seto, Akifumi Onishi, Hitoshi Sugiyama, Norio Sakai, Kenji Yamabe, Yukio Yokoyama, Satoshi Yamashita, Hiroshi Satoh, Shigeru Toyoda, Michihiro Hosojima, Yumi Ito, Ryushi Tazawa, Satoshi Ishii

**Affiliations:** 1 Department of Clinical Nephroscience, Niigata University Graduate School of Medical and Dental Sciences, Niigata 951-8510, Japan; atsut@med.niigata-u.ac.jp (A.T.); m-mikame@med.niigata-u.ac.jp (M.M.); 2 School of Health Sciences, Shinshu University, Matsumoto 390-8621, Japan; izawa611@shinshu-u.ac.jp; 3 Department of Nephrology, Faculty of Medicine, University of Tsukuba, Tsukuba 305-8575, Japan; morito@md.tsukuba.ac.jp; 4 Department of Pediatrics, Yao Municipal Hospital, Yao 581-0069, Japan; swqds602@ybb.ne.jp; 5 Department of Medical Genetics, Osaka City University Graduate School of Medicine, Osaka 545-8585, Japan; setot@med.osaka-cu.ac.jp; 6 Department of Internal Medicine, Fukuyama City Hospital, Fukuyama 721-8511, Japan; onishi10250@fchp.jp; 7 Department of Human Resource Development of Dialysis Therapy for Kidney Disease, Okayama University Graduate School of Medicine, Dentistry and Pharmaceutical Science, Okayama 700-8558, Japan; hitoshis@okayama-u.ac.jp; 8 Child Healthcare and Genetic Science Laboratory, Division of Health Sciences, Osaka University Graduate School of Medicine, Suita 565-0871, Japan; norio@sahs.med.osaka-u.ac.jp; 9 Department of Cardiology, Toyooka Hospital, Toyooka 668-8501, Japan; kenji-yamabe@toyookahp-kumiai.or.jp; 10 Division of Nephrology, Hiroshima Red Cross Hospital & Atomic-bomb Survivors Hospital, Hiroshima 730-8619, Japan; ykykym-srn16@umin.net; 11 Department of Cardiology, Hamamatsu University School of Medicine, Hamamatsu 431-3192, Japan; doug1031@hama-med.ac.jp; 12 Department of Cardiology, Fujinomiya City Hospital, Fujinomiya 418-0076, Japan; satoh36@hospital.fujinomiya.shizuoka.jp; 13 Department of Cardiovascular Medicine, Dokkyo Medical University, Mibu 321-0293, Japan; s-toyoda@dokkyomed.ac.jp; 14 Department of Clinical Nutrition Science, Kidney Research Center, Niigata University Graduate School of Medical and Dental Sciences, Niigata 951-8510, Japan; hoso9582@med.niigata-u.ac.jp; 15 Department of Health Promotion Medicine, Niigata University Graduate School of Medical and Dental Sciences, Niigata 951-8510, Japan; yumii@med.niigata-u.ac.jp; 16 Health Administration Center, Student Support and Health Administration Organization, Tokyo Medical and Dental University, Bunkyo-ku 113-8510, Japan; ryushi.hsc@tmd.ac.jp; 17 GlycoPharma Corporation, Oita 870-0822, Japan; ishiis01@oita-u.ac.jp

**Keywords:** globotriaosylsphingosine, late-onset biopsy-proven Fabry disease, saposin, gene analysis, aberrant splicing transcript

## Abstract

Fabry disease is an X-linked disorder of α-galactosidase A (GLA) deficiency. Our previous interim analysis (1 July 2014 to 31 December 2015) revealed plasma globotriaosylsphingosine as a promising primary screening biomarker for Fabry disease probands. Herein, we report the final results, including patients enrolled from 1 January to 31 December 2016 for evaluating the potential of plasma globotriaosylsphingosine and GLA activity as a combined screening marker. We screened 5691 patients (3439 males) referred from 237 Japanese specialty clinics based on clinical findings suggestive of Fabry disease using plasma globotriaosylsphingosine and GLA activity as primary screening markers, and *GLA* variant status as a secondary screening marker. Of the 14 males who tested positive in the globotriaosylsphingosine screen (≥2.0 ng/mL), 11 with low GLA activity (<4.0 nmol/h/mL) displayed *GLA* variants (four classic, seven late-onset) and one with normal GLA activity and no pathogenic variant displayed lamellar bodies in affected organs, indicating late-onset biopsy-proven Fabry disease. Of the 19 females who tested positive in the globotriaosylsphingosine screen, eight with low GLA activity displayed *GLA* variants (six classic, two late-onset) and five with normal GLA activity displayed a *GLA* variant (one classic) and no pathogenic variant (four late-onset biopsy-proven). The combination of plasma globotriaosylsphingosine and GLA activity can be a primary screening biomarker for classic, late-onset, and late-onset biopsy-proven Fabry disease probands.

## 1. Introduction

Fabry disease (FD; OMIM#301500) is an X-linked lysosomal storage disorder that results from deficient α-galactosidase A (GLA) activity [1]. The major causes of premature morbidity and mortality in patients with FD are progressive cardiac, renal, and cerebrovascular disease [2]. FD can be classified as classic or late-onset [3], which are distinguished by early-onset classic manifestations of acroparesthesia, clustered angiokeratoma, and cornea verticillata in the former, and exclusively cardiac, renal, and cerebrovascular impairments in the latter.

GLA deficiency causes systemic lysosomal accumulation of glycolipids, predominantly globotriaosylceramide (Gb3). The plasma level of the deacylated form of Gb3, globotriaosylsphingosine (lyso-Gb3), was previously identified as a highly reliable biomarker of FD based on studies with confirmed FD patients [4,5]. A gender-specific algorithm for diagnosis using lyso-Gb3 was proposed based on a retrospective study in patients with signs suggestive of FD [6]: GLA activity as a first-tier screen, with *GLA* analysis and lyso-Gb3 level measurement as a second-tier screen was recommended for males, whereas concurrent testing of lyso-Gb3 level and *GLA* analysis was recommended for females. Genetic screening is stressful for patients and medical professionals while imposing high costs and labor. Thus, this algorithm may be unsuitable for screening in a high-risk female population [6].

We have been assessing the value of lyso-Gb3 as a biomarker for screening patients with signs suggestive of FD [7]. Lyso-Gb3 levels are measured by ultra-performance liquid chromatography/tandem mass spectrometry, and plasma GLA activity is measured using the artificial substrate 4-methylumbelliferyl-α-d-galactoside in all patients. This was the first prospective study to clarify that lyso-Gb3 could be a primary screening biomarker to identify probands of both genders among patients with clinical signs suggestive of FD [7]. Based on these interim results, plasma lyso-Gb3 levels should be used in the selection of candidates who may benefit from further *GLA* analysis to improve the outcomes of diagnosis in multispecialty clinics and avoid unnecessary genetic tests [7].

Generally, patients with a normal lyso-Gb3 level and low GLA activity have only class 2 (non-pathogenic) variants [8], including p.Glu66Gln [9] and c.−10C>T [10]. In such cases, simply measuring the lyso-Gb3 level in patients with confirmed low GLA activity can avoid unnecessary *GLA* analysis. Indeed, all FD patients identified in our interim study had positive results in the lyso-Gb3 test [7]. Therefore, it would be considered unnecessary to further measure GLA activity to screen for class 1 (pathogenic) variants [8]. Nevertheless, we encountered a situation in which measuring GLA activity had significant clinical value [7]. Only females suffering from non-specific FD signs (cardiac or renal disease) showed characteristic storage in the affected organs (e.g., the heart and kidney) by electron microscopy, along with high plasma lyso-Gb3 levels but normal GLA activity and no class 1 variant [7]. We speculated that the normal GLA activity detected in such cases may be indicative of the existence of late-onset biopsy-proven FD due to an unknown cause [11]. The identification of male patients with the same condition would lend further support to this idea, demonstrating that the previously proposed gender-specific algorithm [6] may be unsuitable for identifying male late-onset biopsy-proven FD.

GLA in lysosomes hydrolyzes the sphingolipid Gb3 with the help of the sphingolipid activator protein saposin B [12]. The system used for measuring plasma GLA activity evaluates the ability of GLA enzymes to hydrolyze an artificial substrate, 4-methylumbelliferyl-α-d-galactoside, without the use of saposin B. Therefore, saposin B dysfunction is not reflected in this measurement system. Saposin B deficiency has been associated with high plasma lyso-Gb3 levels [13] and accumulation of Gb3 in fibroblasts [13,14]. Therefore, a screening strategy using the combination of the lyso-Gb3 level and GLA activity should also consider the potential of detecting saposin B deficiency.

Although the results of our interim study revealed lyso-Gb3 to be a promising primary screening biomarker for classic and late-onset FD probands [7], only patients with class 2 variants were detected, and FD probands were not identified among patients that experienced early-onset stroke or transient ischemic attack [7]. Recent elucidation of genotype–phenotype relationships of FD [15] revealed that some *GLA* variants previously thought to be class 1 were, in fact, class 2. Therefore, the class 1 variants reported in previous screening studies need to be reinterpreted. Importantly, it remains unclear whether it is reasonable to view patients with early-onset stroke or transient ischemic attack as high-risk targets for screening.

Herein, considering the above context, we present the additional results from patients enrolled in the study from January 1 to 31 December 2016 for confirmation of the diagnosis of FD probands that was established by the interim study [7], and propose an improved screening strategy based on the results from the entire study period.

## 2. Materials and Methods

### 2.1. Lyso-Gb3 and GLA Screening

This prospective multicenter study involved 4 steps: (1) Patients were screened for FD by measuring the plasma lyso-Gb3 level (≥2.0 ng/mL = positive) and plasma GLA activity (<4.0 nmol/h/mL = low); (2) patients with elevated lyso-Gb3 or low GLA activity from step (1) were informed that they may have FD; (3) with the consent of the patient, the FD diagnosis was confirmed by *GLA* analysis; and (4) a familial diagnosis was confirmed by screening other family members based on the lyso-Gb3 level, GLA activity, and *GLA* analysis when indicated.

### 2.2. Patient Enrollment

Japanese patients (aged 0–101 years) of unknown FD status who had been referred by 237 specialty clinics owing to suggestive signs were screened for FD between 1 July 2014 and 31 December 2016.

Clinical evaluations included: cardiac evaluation, including an electrocardiogram, echocardiogram, and cardiac magnetic resonance imaging (for unexplained left ventricular hypertrophy or unexplained cardiac failure); nephrological evaluation with the biochemical examination, urinalysis, imaging, and kidney biopsy (for chronic kidney disease, unexplained proteinuria, or pathological findings consistent with FD); neurological evaluation with magnetic resonance imaging (early-onset stroke or transient ischemic attack); and pediatric evaluation in children with early-onset classic manifestations—acroparesthesia, clustered angiokeratoma, cornea verticillata, and hypohidrosis [7]. Acroparesthesia is defined as pain in the hands and/or feet, with an onset of pain in childhood or adolescence, and/or a course characterized by exacerbations that are provoked by fever, exercise, or heat, as well as a decreased cold sensation [11]. Clustered angiokeratoma should be present in the bathing trunk, periumbilical, and/or perioral regions. Cornea verticillata should be evaluated using a slit lamp in the absence of amphiphilic drug use. Hypohidrosis is defined as low or no sweating even in an environment (high temperature or humidity) that encourages sweating [11]. Patients with known FD and their relatives were excluded.

The study was conducted according to the guidelines of the Declaration of Helsinki and approved by the Ethics Committee of Niigata University School of Medicine (protocol code 1802, 2367, H25–661-627, H27–805-767).

### 2.3. Sample Collection

Blood specimens for the primary screening were collected in Venoject II collection tubes (Terumo, Tokyo, Japan) and immediately refrigerated. Plasma specimens were obtained by centrifugation of whole blood at 1670× *g* for 10 min using a KS-5200C centrifuge (Kubota, Tokyo, Japan) in a refrigerated room at 4 °C and stored frozen at −20 °C until measurement.

### 2.4. Measurement of Plasma Lyso-Gb3

The plasma lyso-Gb3 level was measured by ultra-performance liquid chromatography/tandem mass spectrometry using further purified lyso-Gb3 (Sigma-Aldrich, St. Louis, MO, USA) and a glycine derivative of lyso-Gb3 as the standard and internal standard, respectively. The assays were performed at GlycoPharma (Oita, Japan) with a detection limit of 0.01 ng/mL, as described previously [7]. In a previous study, the cut-off for lyso-Gb3 levels was determined to be 0.9 ng/mL (95th percentile of healthy individuals), with the highest normal value detected at 2.0 ng/mL [16]. To avoid false-positive results, we set the cut-off for lyso-Gb3 levels at 2.0 ng/mL. We evaluated the suitability of this cut-off value for both genders; the highest lyso-Gb3 level among 3400 males with a negative lyso-Gb3 test (<2.0 ng/mL) and normal GLA activity in this study was 1.97 ng/mL, demonstrating the suitability of the 2.0 ng/mL cut-off.

### 2.5. Measurement of Plasma GLA Activity

Plasma GLA activity was also measured at GlycoPharma using the artificial substrate 4-methylumbelliferyl-α-d-galactoside with a detection limit of 0.1 nmol/h/mL, as described previously [7]. To avoid false-negative results, the screening cut-off for the GLA activity level—the percentage of the control mean or cohort mean—was set at ≥50% based on our previous study [7]. We did not establish a reference population for plasma GLA activity; therefore, reference values were not obtained. The cut-off for a low result for plasma GLA activity was set at 4.0 nmol/h/mL, which was 52.0% of the mean cohort value in our interim study [7].

### 2.6. Genetic Counseling

Patients positive in the lyso-Gb3 test or showing low GLA activity values were considered candidates for genetic counseling. The results of lyso-Gb3 and the GLA activity screening were explained to the patients or family members as appropriate.

### 2.7. Gene Analysis

To obtain DNA and RNA samples, blood specimens were collected in Venoject II collection tubes and PAXgene Blood RNA Tubes (PreAnalytiX, Hombrechtikon, Switzerland), respectively, as described previously [7].

*GLA* variants were detected using a combination of genomic DNA and cDNA sequencing at the Department of Clinical Nephroscience, Niigata University Graduate School of Medical and Dental Sciences, according to our previously reported method [7]. In addition, *PSAP* was analyzed for late-onset biopsy-proven FD probands. The cDNA of *PSAP*, including the coding region of saposin B, was amplified using the primer set: sense (*PSAP*-cDNA1S-RP) 5′-CAGGAAACAGCTATGACCGCTATGTACGCCCTCTTCCTC-3′ and antisense (*PSAP*-cDNA1A-T7) 5′-AATACGACTCACTATAGGCTGGGACCTCGTGCTTCTT-3′ [17]. Sequence variants were described according to Human Genome Variation Society recommendations [18].

### 2.8. Defining FD

FD is classified as classic or late-onset FD based on the presence or absence of early-onset classic manifestations and a class 1 variant. Late-onset biopsy-proven FD is defined as late-onset FD with characteristic electron microscopic findings but without a class 1 variant [11].

### 2.9. Statistical Analyses

The data distributions were examined using the Shapiro–Wilk test for *n* ≤ 2000 or using the Kolmogorov–Smirnov–Lillefors test for *n* > 2000 to determine whether they showed a normal distribution. Normally distributed data were assessed for variance using the *F* test. A Student’s *t*-test was used for comparisons between two unpaired groups with homogeneous variances. Welch’s *t*-tests were applied for comparisons between 2 unpaired groups with heterogeneous variances. The Wilcoxon rank-sum test was used for comparison between two unpaired groups when the data were not normally distributed. Values were considered statistically significant at *p* < 0.05. Data were statistically analyzed in JMP^®^12 (SAS Institute, Cary, NC, USA) and graphed in SigmaPlot 14 (Systat Software, San Jose, CA, USA). The receiver operating characteristic (ROC) curve and the area under the curve (AUC) was analyzed and generated in SigmaPlot 14.

## 3. Results

### 3.1. Study Population

A total of 5691 patients (3439 males and 2252 females) from 237 clinics with validated data were enrolled between 2014 and 2016 (Table 1).

### 3.2. Plasma Lyso-Gb3 Levels

Based on plasma lyso-Gb3 levels, patients were classified as positive (≥2.0 ng/mL) or negative (<2.0 ng/mL). The median lyso-Gb3 levels were 14.6 ng/mL [interquartile range (IQR) 4.1–173.5] in the 14 males in the positive group and 0.4 ng/mL (IQR 0.2–0.6) in the 3425 males in the negative group; this difference was significant (Wilcoxon rank-sum test, *P* < 0.0001). The median lyso-Gb3 levels were 15.0 ng/mL (IQR 7.8–21.8) for the 19 females in the positive group and 0.4 ng/mL (IQR 0.3–0.6) for the 2233 females in the negative group; this difference was also significant (Wilcoxon rank-sum test, *P* < 0.0001).

### 3.3. Plasma GLA Activity

The median plasma GLA activity in the 14 males in the positive group was 0.9 nmol/h/mL (IQR 0.4–1.6), which was significantly lower (Wilcoxon rank-sum test, *P* < 0.0001) than the median of 8.1 nmol/h/mL (IQR 6.8–9.6) in the 3425 males in the negative group. GLA activity was low in 13 males in the positive group and in 26 males in the negative group. One male in the positive group had normal GLA activity. The median GLA activity in the 19 females in the positive group was 3.0 nmol/h/mL (IQR 1.9–5.9), which was significantly lower (Wilcoxon rank-sum test, *P* < 0.0001) than that in the 2233 females in the negative group of 7.4 nmol/h/mL (IQR 6.2–8.8). Eight females in the positive group had normal GLA activity and 21 females in the negative group had low GLA activity.

### 3.4. Classification of FD Probands with Class 1 Variants

**Figure 1:**
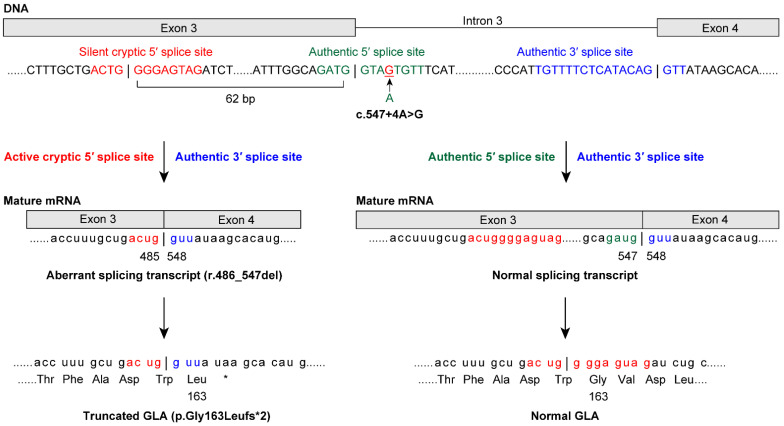
The 5′splice site (5′ss) variant c.547+4A>G causes normal and aberrant splicing in a male patient with late-onset Fabry disease (FD). A novel *GLA* variant was identified at the intronic site of the 5′ss at positions +3 to +6. Genomic sequencing revealed the c.547+4A>G variant, which was predicted to result in aberrant splicing using the SD-Score algorithm—a practical tool to predict splicing consequences of variants at the 5′ss [31]. cDNA sequencing further revealed that the authentic 5′ss was partially inactivated. The 5′ss variant acted as the authentic 5′ss and produced a normal transcript and GLA protein. Moreover, the variant activated the cryptic 5′ss at position −62 in exon 3. This was consistent with a report showing that almost all of the major cryptic sites activated by variants are mapped within an approximate 100-nucleotide region from the authentic 5′ss [32]. The activated cryptic 5′ss caused aberrant splicing outside the GT-AG rule [33], and the activated cryptic 5′ss produced the aberrant transcript (r.486_547del62) and the truncated GLA protein (p.Gly163Leufs*2). Indeed, both the 5′ss and activated cryptic 5′ss were identified as candidates for 5′ss using GENETYX Ver.13 (Genetyx, Tokyo, Japan). In cryptic site activation, the authentic site is not always completely inactivated [32]. This mechanism explains late-onset FD in patients with the c.547+4A>G variant.

Among the patients enrolled in the study, *GLA* analysis revealed that 20 of the FD probands (11 males and nine females) had class 1 variants (Table 2). Among the cases of FD identified in this study, we found 15 previously reported *GLA* (NM_000169.2) variants [c.44C>G p.(Ala15Gly) [19], c.(202C>T) p.Leu68Phe [20], c.(281G>A) p.Cys94Tyr [21], c.334C>T p.(Arg112Cys) [21], c.(335G>A) p.Arg112His [22], c.(658C>T) p.Arg220* [21], c.(659G>C) p.Arg220Pro [23], c.691G>A p.Asp231Asn [24], c.[788A>G];[0] p.(Asn263Ser) [25], c.(902G>A) p.Arg301Gln [26], c.(935A>G) p.Gln312Arg [27], c.1133G>T p.Cys378Phe [28], c.1171A>G p.(Lys391Glu) [29], c.1208T>C p.(Leu403Ser) [27], and c.1244T>C p.(Leu415Pro) [30]]. Four novel variants were identified: a splicing variant, NG_007119.1(NM_000169.2): c.547+4A>G [normal transcript from splicing at the authentic 5′ splice site (5′ss) and an aberrant splicing transcript r.486_547del, p.Gly163Leufs*2 from splicing at the activated cryptic 5′ss in exon 3; Figure 1], c.(254G>T) p.Gly85Val, c.559_560del p.(Met187Valfs*6), and c.1163T>A p.(Leu388His). Thus, lyso-Gb3 screening identified both classic and late-onset FD probands in males and females.

Among the patients enrolled in 2016, *GLA* analysis revealed that seven of the FD probands (four males and three females) had class 1 variants. All of the male FD probands were in the positive group and had low GLA activity. Both of the male classic FD probands were pediatric patients; of the two late-onset FD probands, one was referred from cardiology and the other from nephrology. All of the female FD probands were in the positive group and all had low GLA activity. Of the two female classic FD probands, one was referred from cardiology and the other from nephrology. There was one female late-onset FD proband referred from nephrology.

### 3.5. Frequency of Positive Lyso-Gb3 Screens and FD Diagnosis by Clinical Department

In all clinical departments, except for pediatrics, the frequency of identification among patients who screened positive for lyso-Gb3 was higher in females than in males (Table 3). Of the referring departments that treated adult patients, cardiology had the highest frequency among females who screened positive for lyso-Gb3 (Table 3). In the neurological department, only one female proband was found to have classic FD with acroparesthesia but no signs of early-onset stroke [7]. However, no FD proband was identified among early-onset stroke patients in both the interim study [7] and the present study.

### 3.6. Prosaposin (PSAP) Analysis for Probands with Late-Onset Biopsy-Proven FD

Five patients (one male and four females) had pathological findings suggestive of FD by biopsy. Surprisingly, plasma GLA activity was normal, although lyso-Gb3 levels were high in all patients. Subsequent *GLA* analysis showed no class 1 variants. A diagnosis of late-onset biopsy-proven FD probands was made for three females in the interim analysis (Table 4) [7] and for one male and one female, each enrolled in 2016 (Table 4). Additionally, no variants in the coding region of saposin B were identified in *PSAP* (NM_002778.3) analysis for these probands.

### 3.7. Comparison of Lyso-Gb3 Screening and GLA Analysis between Cohorts

**Figure 2:**
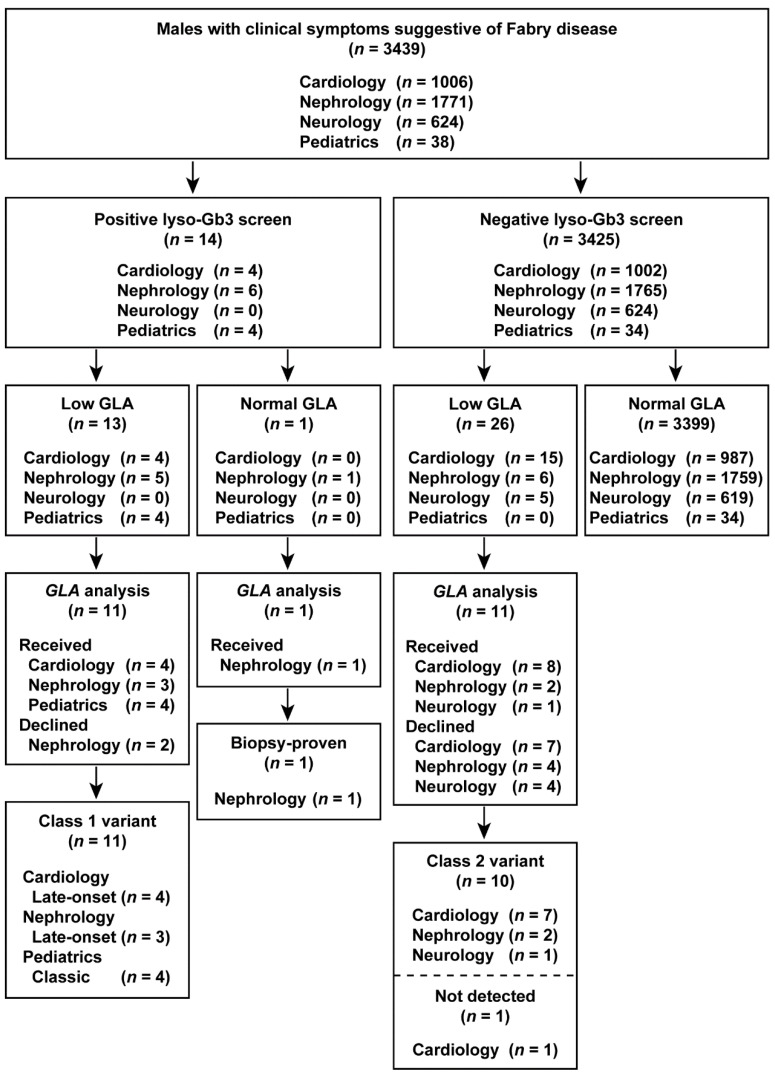
Flowchart of the combination of lyso-Gb3 and GLA activity screening and *GLA* analysis in male patients. Male patients enrolled between 1 July 2014 and 31 December 2016.

**Figure 3:**
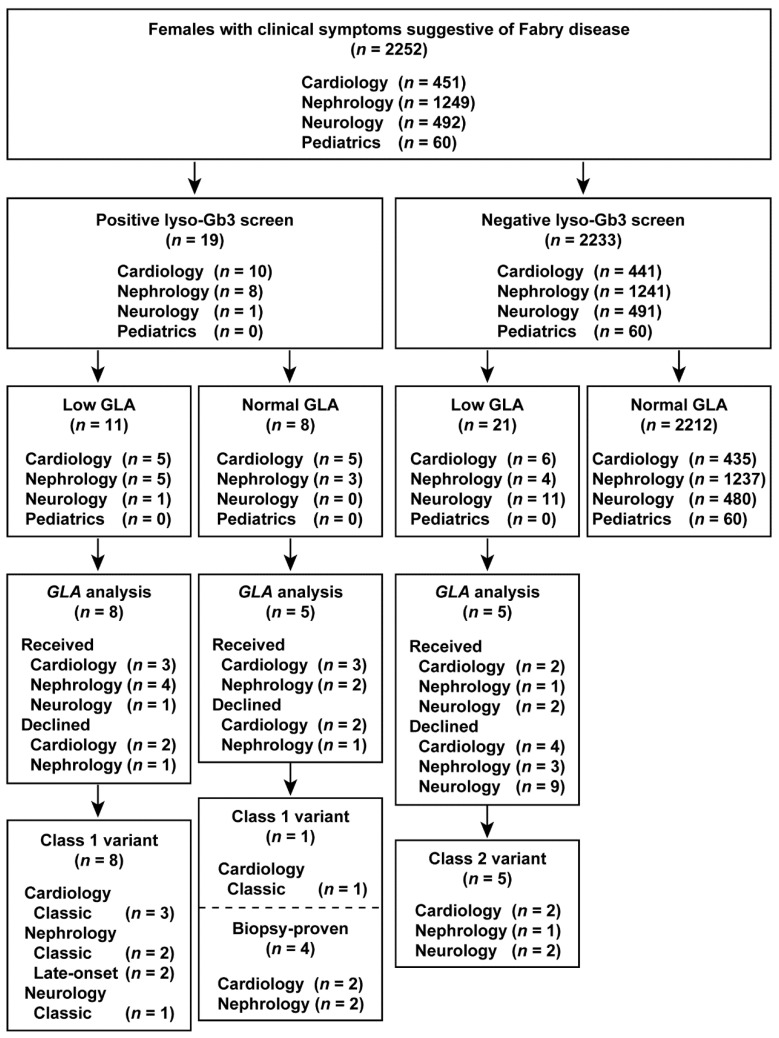
Flowchart of the combination of lyso-Gb3 and GLA activity screening and *GLA* analysis in female patients. Female patients enrolled between 1 July 2014 and 31 December 2016.

The results of the study are summarized in flowcharts in Figure 2 and Figure 3. Overall, we identified classic, late-onset, and late-onset biopsy-proven FD probands in both males and females who screened positive for elevated lyso-Gb3 levels.

### 3.8. Differences in Plasma Lyso-Gb3 Levels and GLA Activity between Cohorts

**Figure 4:**
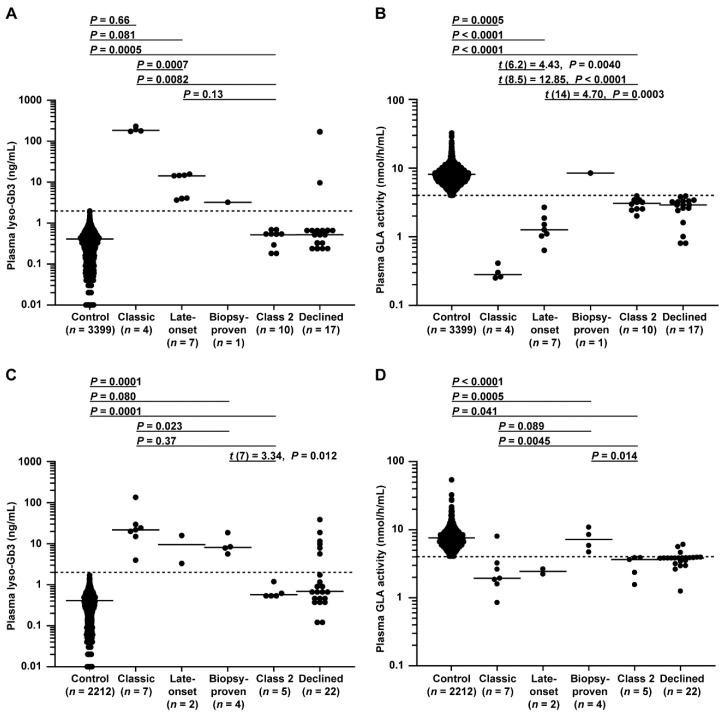
Plasma lyso-Gb3 levels and GLA activity in male and female patients. (**A**,**B**) Male patients enrolled between 1 July 2014 and 31 December 2016 belonging to control, classic, late-onset, late-onset biopsy-proven, class 2, or declined groups; patients belonging to the late-onset biopsy-proven group, which was unsuitable for statistical analysis due to its small number, not detected group (*n* = 1) summarized in flowcharts in Figure 2, and declined group, in which *GLA* variants were not analyzed, were excluded from the analysis. (**A**) The dotted line indicates the threshold for a positive test of 2.0 ng/mL. Short horizontal lines indicate the median plasma lyso-Gb3 value in each group. When plasma lyso-Gb3 values were less than the detection limit of the assay (0.01 ng/mL), a value of 0 ng/mL was used to represent the lyso-Gb3 levels in the statistical analysis. Patients with a lyso-Gb3 value of 0 (*n* =156 males in the control and *n* = 1 male in the class 2 group in **A**) could not be plotted on a logarithmic graph. (**B**) The dotted line indicates the threshold of 4.0 nmol/h/mL for a positive test. Short horizontal lines indicate the median plasma GLA activity in each group. Plasma GLA activities were detected in all patients. Differences between groups were evaluated with the Student’s *t*-test, Welch’s *t*-test, or Wilcoxon rank-sum test; data are shown as *t* (integral degree of freedom) = *t* value and *P* value (Student’s *t*-test); *t* (mixed decimal degree of freedom) = *t* value and *P* value (Welch’s *t*-test); or *P* value only (Wilcoxon rank-sum test). (**C**,**D**) Female patients enrolled between July 1 2014 and 31 December 2016 belonging to control, classic, late-onset, late-onset biopsy-proven, class 2, or declined groups; patients belonging to the late-onset group, which was unsuitable for statistical analysis due to its small number, and declined group, in which *GLA* variants were not analyzed, were excluded from the analysis. (**C**) The dotted line is the threshold for a positive test of 2.0 ng/mL. Short horizontal lines indicate the median plasma lyso-Gb3 value in each group. When plasma lyso-Gb3 values were less than the detection limit of the assay (0.01 ng/mL), a value of 0 ng/mL was used to represent the lyso-Gb3 levels in the statistical analysis. Patients with a lyso-Gb3 value of 0 (*n* = 123 in the control in **C**) could not be plotted on a logarithmic graph. (**D**) The dotted line indicates the threshold of 4.0 nmol/h/mL. Short horizontal lines indicate the median plasma GLA activity in each group. Plasma GLA activities were detected in all patients. Differences between groups were evaluated with Student’s *t*-test or the Wilcoxon rank-sum test; data are shown as *t* (integral degree of freedom) = *t* value and *P* value (Student’s *t*-test) or *P* value only (Wilcoxon rank-sum test).

Plasma lyso-Gb3 levels and GLA activity in male patients are shown in Figure 4A, B. There were significant differences between FD probands and controls in these markers. Significant differences in these markers were also detected between classic and late-onset FD probands. Specifically, the lyso-Gb3 level differed between late-onset biopsy-proven FD and controls, and the GLA activity differed between late-onset biopsy-proven FD and late-onset FD probands.

Plasma lyso-Gb3 levels and GLA activity in female patients are shown in Figure 4C,D. Similar to the results for the males, there were significant differences found between FD probands and controls in these markers. Late-onset biopsy-proven FD probands showed higher lyso-Gb3 levels than controls and a higher trend in GLA activity than that of late-onset FD probands.

Importantly, these results suggested that a form of late-onset biopsy-proven FD may exist with a distinct FD pathophysiology. As expected, no class 2 variant could be detected based on lyso-Gb3 alone in both sexes.

### 3.9. ROC Curve Analysis for Plasma Lyso-Gb3 Levels, GLA Activity, and GLA/lyso-Gb3 Ratio

The GLA/lyso-Gb3 ratio was recently suggested as a promising biomarker for female FD patients [34]. Therefore, we assessed the suitability of lyso-Gb3 levels, GLA activity, and the GLA/lyso-Gb3 ratio as a biomarker for screening probands. To compare the ROC curves and AUC values of these biomarkers in the same graph, instead of lyso-Gb3 levels, we used the reciprocal (1/lyso-Gb3 levels), which were low in FD probands. In both sexes, 1/lyso-Gb3 levels and the GLA/lyso-Gb3 ratio emerged as more sensitive and specific biomarkers than GLA activity (Supplementary Figure S1A–D). There were no differences in the sensitivity or specificity of 1/lyso-Gb3 levels and the GLA/lyso-Gb3 ratio for discriminating FD (Supplementary Figure S1A–D).

## 4. Discussion

We assessed the applicability of a combination of plasma lyso-Gb3 and GLA activity as a biomarker for FD screening in male and female patients with signs suggestive of FD. This method enabled uncovering and discriminating among classic, late-onset, and late-onset biopsy-proven FD probands. Moreover, the existence of late-onset biopsy-proven FD as a pathologically distinct entity has been revealed.

Herein, late-onset biopsy-proven FD was exclusively identified in male and female patients with elevated plasma lyso-Gb3 levels and normal GLA activity. Saposin B deficiency, which presents with similar plasma findings, was excluded as the cause based on *PSAP* analysis. Nevertheless, the differences, if any, between late-onset biopsy-proven FD and late-onset FD remain unclear, along with the appropriate therapy for the former category. Thus, it is important to clarify the pathophysiology of late-onset biopsy-proven FD.

Considering the X-linked hereditary form of FD, the incidence of FD in females (with two X chromosomes) should be about double that of males (with one X chromosome) [35]. Nonetheless, fewer female patients were detected than male patients, and the female patients were more often diagnosed with classic than with late-onset FD, suggesting that our screening strategy may be insufficient for detecting female late-onset FD patients. First, female late-onset FD patients who are less symptomatic than classic FD patients may be less likely to be screened even if they have elevated lyso-Gb3 levels. Second, the cut-off value may be high for screening in female late-onset FD patients. Third, most female late-onset FD patients may have normal lyso-Gb3 levels.

A strategy of a primary screen by GLA activity and a secondary screen by the lyso-Gb3 level may not detect late-onset biopsy-proven FD or some female FD patients with a negative primary screen result because of normal GLA activity. In contrast, our primary screening strategy using concurrent measurements of plasma lyso-Gb3 level and GLA activity may detect these cases. Moreover, *GLA* analysis imposes stress on the medical professional who will suggest and perform the test for the patient [36]. Therefore, a lyso-Gb3 test result that can accurately suggest the need for *GLA* analysis and supports the results of *GLA* analysis will help to reduce the stress on the medical professional.

Most variants of the 5′ss were observed at the intronic GT site at positions +1 and +2 [37]. In the present study, a novel *GLA* variant was identified at the intronic site of the 5′ss at positions +3 to +6 (Figure 1). Genomic sequencing revealed c.547+4A>G, which was predicted to result in aberrant splicing using the SD-Score algorithm [31]. cDNA sequencing revealed that the authentic 5′ss was partially inactivated, acting as the authentic 5′ss and producing a normal transcript and GLA protein. Moreover, this variant activated the cryptic 5′ss at position −62 in exon 3, causing aberrant splicing outside the GT-AG rule [33], and the cryptic 5′ss produced an aberrant transcript (r.486_547del) and a truncated GLA protein (p.Gly163Leufs*2). Thus, in cryptic site activation, the authentic site is not always completely inactivated [32]. Similarly, the authentic 5′ss with the c.801+2_801+3insT variant [38] was completely inactivated and produced a transcript with the entire 217-bp intron 5 and T insertion, and p.Leu268Valfs*27. The cryptic 5′ss at position +36 in intron 5 was activated and produced a transcript with a 36-bp initial region of intron 5 and T insertion, and p.Leu268Valfs*43. Taken together, these results suggest that the function of an authentic 5′ss variant—production of a normal splicing transcript—may be the key to late-onset (c.547+4A>G) or classic (c.801+2_801+3insT) phenotypes due to 5′ss variants. Our method could identify variants in the exon–intron boundary regions as well as in exons. [7,39] Conversely, when no *GLA* variant is found using our method, we consider that further laborious intron analysis would be unnecessary because it only detected genetic variants of uncertain significance [7].

The initial report on screening for FD in young patients with cerebrovascular disease showed a high frequency of detection, but no information about *GLA* variants was provided (Supplementary Table S1) [40]. Unexpectedly, no probands with cerebrovascular disease were detected in our entire study. Recent elucidation of the genotype–phenotype relationship resulted in a change of *GLA* variants from class 1 to class 2 [15]. Only 13 variants were detected (Supplementary Table S1), including only one class 1 variant (p.Arg227Gln) detected in a classic FD patient [41]. Eight of these variants—c.−44C>T [42], c.−10C>T [43], p.Glu66Gln [44], p.Asp83Asn [45], p.Arg118Cys [45], p.Ser126Gly [45], p.Ala143Thr [45], and p.Asp313Tyr [46]—have been interpreted as class 2 variants, and the remaining four variants—p.Ser102Leu [47], p.Val316Ile [47], p.Leu415Phe [47], and p.Glu418Gly [47]—have been interpreted as benign. Importantly, individuals with the variants c.−10C>T [7], p.Glu66Gln [44], p.Asp83Asn [10], p.Arg118Cys [10], p.Ser126Gly [48], p.Ala143Thr [48], and p.Asp313Tyr [48] all showed normal plasma lyso-Gb3 levels. The combination of assessing GLA activity and the lyso-Gb3 level avoided unnecessary *GLA* analysis in patients without FD. Accordingly, young patients with cerebrovascular disease may not be a high-risk population for FD.

This study has several limitations. First, 39 patients with signs suggestive of FD declined further *GLA* analysis; therefore, some diagnoses of FD may have been missed. However, the decisions of patients must be respected. Second, late-onset biopsy-proven FD was more easily detected than late-onset FD in females, suggesting that a disease other than FD might have been detected.

Patients tend to experience psychological stress during the process of screening for a diagnosis of FD. This process can be the first step in coming to terms with FD concerns that the patient may encounter later. Therefore, our careful procedures during screening will help patients successfully navigate this step.

In conclusion, the combination of plasma lyso-Gb3 and GLA activity can be a primary screening biomarker for classic, late-onset, and late-onset biopsy-proven FD probands. The future research avenue is the evolution of our screening strategy to detect female patients with late-onset FD more successfully. Moreover, future research will focus on understanding the pathogenesis of late-onset biopsy-proven FD.

## Figures and Tables

**Table 1:** Patients grouped according to the medical specialty of the referring clinic.

	Cardiology	Nephrology	Neurology	Pediatrics	Total
**Males**	**(*n* = 1006)**	**(*n* = 1771)**	**(*n* = 624)**	**(*n* = 38)**	**(*n* = 3439)**
Median (IQR) age (years)	64 (51–71)	66 (56–74)	54 (47–71)	11 (9–13)	64 (51–73)
Clinics (*n*)	59	47	43	13	162
**Females**	**(*n* = 451)**	**(*n* = 1249)**	**(*n* = 492)**	**(*n* = 60)**	**(*n* = 2252)**
Median (IQR) age (years)	67 (53–75)	69 (61–77)	66 (47–81)	12 (9–14)	68 (54–77)
Clinics (*n*)	74	57	57	26	214

**Table 2:** Characterization of patients with Fabry disease.

	Patient No.	Lyso-Gb3 Levels (ng/mL)	GLA Activity (nmol/h/mL)	*GLA* Variants		Age (Years)	Early-Onset Classic Manifestations	Manifestations		
				DNA	Protein			Heart	Kidneys	CNS
**Males; Classic type**										
Pediatrics	1 ^1^	190.2	0.4	c.(202C>T)	p.Leu68Phe	13	Acroparesthesia Hypohidrosis	None	None	None
	2 ^1^	172.2	0.3	c.(254G>T)	p.Gly85Val	9	Acroparesthesia Hypohidrosis	None	None	None
	3	230.1	0.3	c.(658C>T)	p.Arg220*	9	Acroparesthesia Hypohidrosis	None	None	None
	4	177.4	0.3	c.1133G>T	p.Cys378Phe	9	Acroparesthesia Hypohidrosis	None	None	None
**Males; Late-onset type**										
Cardiology	5 ^1^	3.6	2.7	c.(335G>A)	p.Arg112His	61	None	LVH	G5DA1	None
	6	14.2	1.5	c.547+4A>G r.486_547del	p.Gly163Leufs*2	74	None	Arrhythmia LVH	G3a	None
	7 ^1^	15.6	1.1	c.(902G>A)	p.Arg301Gln	55	None	LVH	None	None
	8 ^1^	14.5	1.0	c.(935A>G)	p.Gln312Arg	77	None	LVH	None	None
Nephrology	9	14.7	1.9	c.44C>G	p.(Ala15Gly)	66	None	Arrhythmia LVH	G3A2	None
	10 ^1^	4.1	0.6	c.(335G>A)	p.Arg112His	42	None	None	G2A3	None
	11 ^1^	4.0	1.3	c.1171A>G	p.(Lys391Glu)	75	None	None	G5DA2	Stroke
**Females; Classic type**										
Cardiology	12	29.5	1.9	c.(281G>A)	p.Cys94Tyr	71	None	Arrhythmia LVH	G3a	Stroke
	13 ^1^	21.8	3.2	c.559_560del	p.(Met187Valfs*6)	65	Acroparesthesia Gastrointestinal symptoms	LVH	None	Stroke
	14 ^1^	24.2	1.6	c.(659G>C)	p.Arg220Pro	65	Angiokeratoma Cornea verticillata	LVH	G3bA1	Stroke
	15 ^1^	4.0	8.0	c.691G>A	p.Asp231Asn	63	None	Arrhythmia Heart failure	None	None
Nephrology	16	19.9	1.9	c.334C>T	p.(Arg112Cys)	44	None	LVH	G2A3	None
	17 ^1^	15.0	2.6	c.1244T>C	p.(Leu415Pro)	32	Acroparesthesia	LVH	G1A3	None
Neurology	18 ^1^	135.0	0.8	c.[788A>G];[0]	p.(Asn263Ser)	27	Acroparesthesia Hypohidrosis Angiokeratoma	Arrhythmia	G1A3	None
**Females; Late-onset type**										
Nephrology	19	15.8	2.2	c.1163T>A	p.(Leu388His)	59	None	LVH	G3aA3	None
	20 ^1^	3.3	2.6	c.1208T>C	p.(Leu403Ser)	53	None	LVH	G5A3	None

^1^ Patients analyzed in our interim report [7]. CNS, central nervous system; LVH, left ventricular hypertrophy. Glomerular filtration rate category (mL/min/1.73 m^2^): G1, ≥90; G2, 60–89; G3a, 45–59; G3b, 30–44; G4, 15–29; G5, <15. D, dialysis. Proteinuria category (g/gCr): A1, <0.15; A2, 0.15–0.49; A3, ≥0.50. Patient No.18 was diagnosed as having Turner’s syndrome.

**Table 3:** Frequencies of patients who screened positive for lyso-Gb3 and class 1 *GLA* variants, grouped according to the specialty of the referring clinic.

		Cardiology	Nephrology	Neurology	Pediatrics	Total
Males	Lyso-Gb3-positive patients, *n*/*N* (%)	4/1006 (0.4)	6/1771 (0.3)	0/624 (0)	4/38 (10.5)	14/3439 (0.4)
	*GLA* variant, *n*/*N* (%)	4/1006 (0.4)	3/1771 (0.2)	0/624 (0)	4/38 (10.5)	11/3439 (0.3)
	Classic type, *n*	0	0	0	4	4
	Late-onset type, *n*	4	3	0	0	7
Females	Lyso-Gb3-positive patients, *n*/*N* (%)	10/451 (2.2)	8/1249 (0.6)	1/492 (0.2)	0/60 (0)	19/2252 (0.8)
	*GLA* variant, *n*/*N* (%)	4/451 (0.9)	4/1249 (0.3)	1/492 (0.2)	0/60 (0)	9/2252 (0.4)
	Classic type, *n*	4	2	1	0	7
	Late-onset type, *n*	0	2	0	0	2

**Table 4:** Clinical manifestations in probands with late-onset biopsy-proven Fabry disease.

Department	Patient No.	Lyso-Gb3 Levels (ng/mL)	GLA Activity (nmol/h/mL)	*GLA* Class 1 Variants	Age (years)	Early-Onset Classic Manifestations	Manifestations		
							Heart	Kidneys	CNS
**Male**									
Nephrology	1	3.2	8.4	None	75	None	None	G2A1 Lamellar body	None
**Females**									
Cardiology	2 ^1^	8.4	8.5	None	59	None	Arrhythmia LVH Lamellar body	G5A3 Lamellar body	None
	3 ^1^	18.5	10.9	None	67	None	Arrhythmia LVH Lamellar body	None	None
Nephrology	4 ^1^	7.8	4.7	None	69	None	Arrhythmia	G3aA3 Lamellar body	None
	5	5.6	5.9	None	66	None	None	G3aA3 Lamellar body	None

^1^ Patients analyzed in our interim report [7]. CNS, central nervous system; LVH, left ventricular hypertrophy. Glomerular filtration rate category (mL/min/1.73 m^2^): G2, 60–89; G3a, 45–59; G5, <15. Proteinuria category (g/gCr): A1, <0.15; A3, ≥0.50.

## Data Availability

The data presented in this study are available on request from the corresponding author.

## Supplementary Materials

The following are available online at https://www.mdpi.com/article/10.3390/cimb43010032/s1, Figure S1: Comparison of receiver operating characteristic (ROC) curves for plasma 1/lyso-Gb3 levels, GLA activity, and GLA/lyso-Gb3 ratio for male and female patients enrolled from 2014 to 2016, Table S1: *GLA* variants identified by screening for Fabry disease in young patients with cerebrovascular diseases.
