# Supplementary material for: Plasma Globotriaosylsphingosine and α-Galactosidase A Activity as a Combined Screening Biomarker for Fabry Disease in a Large Japanese Cohort
Source: Curr Issues Mol Biol. 2021 Jun 19;43(1):389–404. doi: 10.3390/cimb43010032 (PMC8928976; doi:10.3390/cimb43010032)
Supplement: Supplementary file 1 [file cimb-43-00032-s001.zip › cimb-1239432-supplementary.pdf]

Plasma Globotriaosylsphingosine and α-Galactosidase A Activity as a Combined Screening Biomarker for Fabry Disease in a Large Japanese Cohort

1. SUPPLEMENTARY FIGURE
Supplementary Figure S1

2. SUPPLEMENTARY TABLE
Supplementary Table S1

3. SUPPLEMENTARY REFERENCES

4. SUPPLEMENTARY ACKNOWLEDGMENTS

1. SUPPLEMENTARY FIGURE

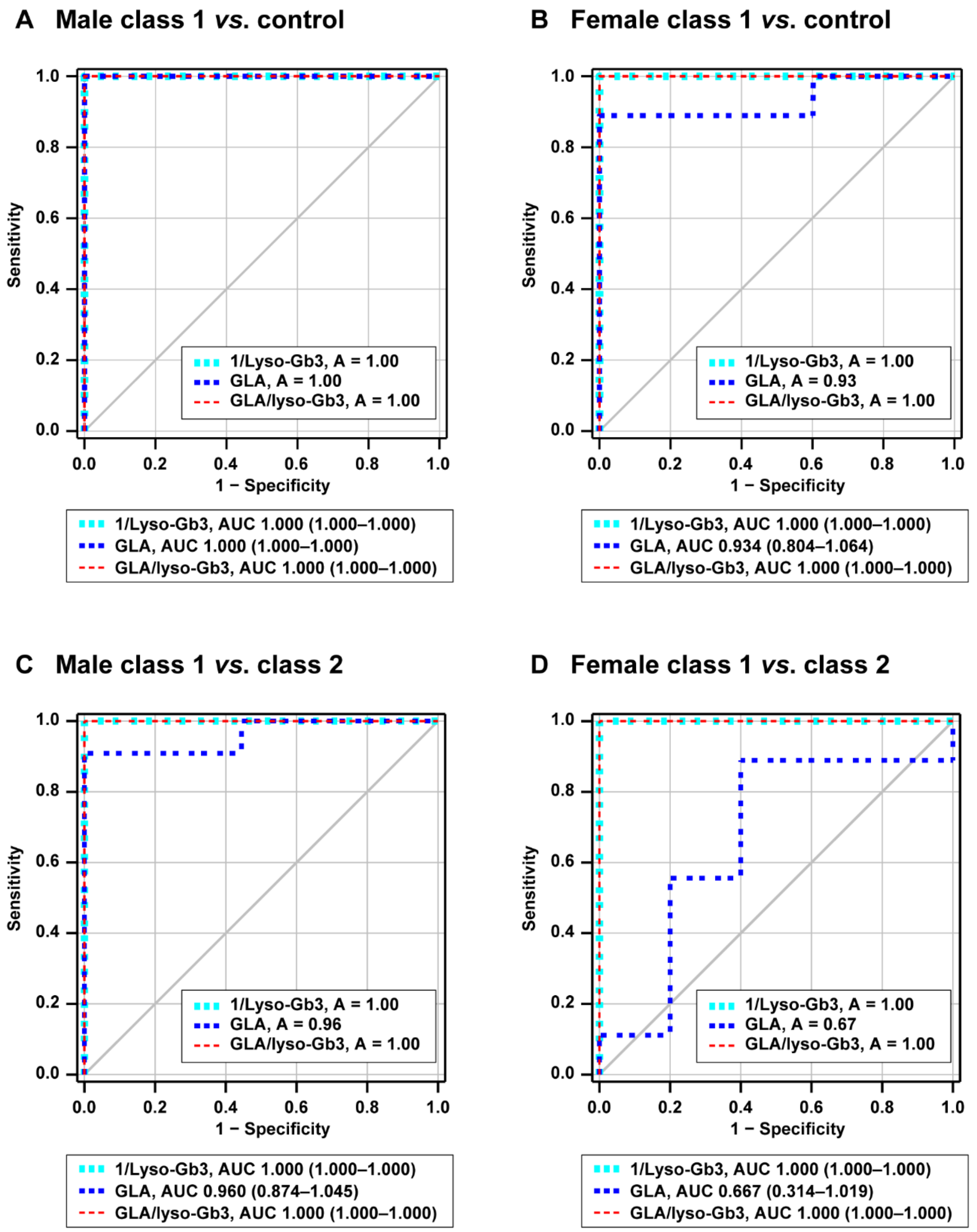

Supplementary Figure S1. Comparison of receiver operating characteristic (ROC) curves for plasma 1/lyso-Gb3 levels, GLA activity, and GLA/lyso-Gb3 ratio for male and female patients enrolled from 2014 to 2016. When plasma lyso-Gb3 values were less than the detection limit of the assay (0.01 nmol/h/mL), a value of 0 ng/mL was used to represent the lyso-Gb3 levels in the statistical analysis. (**A**)

Discrimination between male patients with a class 1 variant (*n* = 11) and controls (*n* = 3243). Because 156 of the 3399 control males had a lyso-Gb3 level of 0 (see Figure 4A), these patients were excluded from the analysis. (**B**) Discrimination between female patients with a class 1 variant (*n* = 9) and controls (*n* = 2089). Because 123 of the 2212 control males had a lyso-Gb3 level of 0 (see Figure 4C), these patients were excluded from the analysis. (**C**) Discrimination between male patients with class 1 (*n* = 11) and class 2 (*n* = 9) variants. Because 1 of the 10 class 2 males had a lyso-Gb3 level of 0 (see Figure 4A), this patient was excluded from the analysis. (**D**) Discrimination between female patients with class 1 (*n* = 9) and class 2 (*n* = 5) variants.

2. SUPPLEMENTARY TABLE

Supplementary Table S1. *GLA* variants identified by screening for Fabry disease in young patients with cerebrovascular diseases.

References (year of publication)	Participants (n) Males	Females	Total	Fabry disease diagnosis (n)	*GLA* variants	Class 1 variants (n)
Rolfs *et al*. (2005)[S1]	432	289	721	28	Not reported	0
Brouns *et al*. (2007)[S2]	64	39	103	0	None	0
Wozniak *et al*. (2010)[S3]	558	0	558	1	p.Ala143Thr (FD-causing)[1]	0
				0	p.Asp313Tyr (polymorphism)[1]	0
Baptista *et al*. (2010)[S4]	300	193	493	2	c.–44C>T	0
				6	p.Arg118Cys	0
				6	p.Asp313Tyr	0
Brouns *et al*. (2010)[S5]	–	–	842	1	p.Ser126Gly[1]	0
				2	p.Ala143Thr[1]	0
				3	p.Asp313Tyr[1]	0
Sarikaya *et al*. (2012)[S6]	102	48	150	0	None	0
Marquardt *et al*. (2012)[S7]	85	69	154	0	None[2]	0
Rolfs *et al*. (2013)[S8]			5023	27[3]		0
					p.Asp83Asn[1]	
					p.Ser102Leu[1]	
					p.Arg118Cys[1]	
					p.Ser126Gly[1]	

					p.Ala143Thr[1]<br>p.Val316Ile[1]<br>p.Leu415Phe[1]<br>p.Glu418Gly[1]	
Dubuc *et al*. (2013)[S9]	55	45	100	1	c.–10C>T, c.370–81_370–77delCAGCC, c.640–16A>G, c.1000–22C>T	0
Fancellu *et al*. (2015)[S10]	–	–	148	1	p.Arg227Gln[1,4]	1
				1	p.Asp313Tyr[1]	0
Lanthier *et al*. (2017)[S11]	218	179	397	1	p.Arg118Cys	0
				0	p.Asp313Tyr (neutral)	0
				0	c.–10C>T, c.370–81_370–77delCAGCC, c.640–16A>G, c.1000–22C>T (neutral)	0
				0	c.1000–22C>T, c.1290+43A>G (neutral)	0
Kinoshita *et al*. (2018)[S12]	394	120	516	0	p.Glu66Gln (non-pathogenic)[1]	0
Maruyama *et al*. (2019)[S13]	281	220	501[5]	0	None	0

The methods of *GLA* analyses:

[1] Only the protein variants were described with no description of the method of gene analysis; therefore, the DNA variants were not classified based on the Human Genome Variation Society recommendations.

[2] Five patients who had the *GLA* variant p.Asp313Tyr were excluded because they were over 60 years old.

[3] The number of each *GLA* variant was not described.

[4] The male proband with classic Fabry disease (p.Arg227Gln) exhibited acroparesthesia, multiple white matter lesions, and severe cardiac and renal involvement.
[5] Patients with cerebrovascular disease referred from neurology.

3. SUPPLEMENTARY REFERENCES

S1. Rolfs, A.; Böttcher, T.; Zschiesche, M.; Morris, P.; Winchester, B.; Bauer, P.; Walter, U.; Mix, E.; Löhr, M.; Harzer, K.; et al. Prevalence of Fabry disease in patients with cryptogenic stroke: a prospective study. *Lancet* **2005**, *366*, 1794–1796, doi:10.1016/S0140-6736(05)67635-0.

S2. Brouns, R.; Sheorajpanday, R.; Braxel, E.; Eyskens, F.; Baker, R.; Hughes, D.; Mehta, A.; Timmerman, T.; Vincent, M.F.; De Deyn, P.P. Middelheim Fabry Study (MiFaS): a retrospective Belgian study on the prevalence of Fabry disease in young patients with cryptogenic stroke. *Clin. Neurol. Neurosurg.* **2007**, *109*, 479–484, doi:10.1016/j.clineuro.2007.03.008.

S3. Wozniak, M.A.; Kittner, S.J.; Tuhrim, S.; Cole, J.W.; Stern, B.; Dobbins, M.; Grace, M.E.; Nazarenko, I.; Dobrovolny, R.; McDade, E.; et al. Frequency of unrecognized Fabry disease among young European-American and African-American men with first ischemic stroke. *Stroke* **2010**, *41*, 78–81, doi:10.1161/STROKEAHA.109.558320.

S4. Baptista, M.V.; Ferreira, S.; Pinho-E-Melo, T.; Carvalho, M.; Cruz, V.T.; Carmona, C.; Silva, F.A.; Tuna, A.; Rodrigues, M.; Ferreira, C.; et al. Mutations of the *GLA* gene in young patients with stroke: the PORTYSTROKE study—screening genetic conditions in Portuguese young stroke patients. *Stroke* **2010**, *41*, 431–436, doi:10.1161/STROKEAHA.109.570499.

S5. Brouns, R.; Thijs, V.; Eyskens, F.; Van den Broeck, M.; Belachew, S.; Van Broeckhoven, C.; Redondo, P.; Hemelsoet, D.; Fumal, A.; Jeangette, S.; et al. Belgian Fabry study: prevalence of Fabry disease in a cohort of 1000 young patients with cerebrovascular disease. *Stroke* **2010**, *41*, 863–868, doi:10.1161/STROKEAHA.110.579409.

S6. Sarikaya, H.; Yilmaz, M.; Michael, N.; Miserez, A.R.; Steinmann, B.; Baumgartner, R.W. Zurich Fabry study—prevalence of Fabry disease in young patients with first cryptogenic ischaemic stroke or TIA. *Eur. J. Neurol.* **2012**, *19*, 1421–1426, doi:10.1111/j.1468-1331.2012.03737.x.

S7. Marquardt, L.; Baker, R.; Segal, H.; Burgess, A.I.; Poole, D.; Hughes, D.A.; Rothwell, P.M. Fabry disease in unselected patients with TIA or stroke: population-based study. *Eur. J. Neurol.* **2012**, *19*, 1427–1432, doi:10.1111/j.1468-1331.2012.03739.x.

S8. Rolfs, A.; Fazekas, F.; Grittner, U.; Dichgans, M.; Martus, P.; Holzhausen, M.; Böttcher, T.; Heuschmann, P.U.; Tatlisumak, T.; Tanislav, C.; et al. Acute cerebrovascular disease in the young: the stroke in young Fabry patients study. *Stroke* **2013**, *44*, 340–349, doi:10.1161/STROKEAHA.112.663708.

S9. Dubuc, V.; Moore, D.F.; Gioia, L.C.; Saposnik, G.; Selchen, D.; Lanthier, S.

Prevalence of Fabry disease in young patients with cryptogenic ischemic stroke. *J. Stroke Cerebrovasc. Dis.* **2013**, *22*, 1288–1292, doi:10.1016/j.jstrokecerebrovasdis.2012.10.005.

S10. Fancellu, L.; Borsini, W.; Romani, I.; Pirisi, A.; Deiana, G.A.; Sechi, E.; Doneddu, P.E.; Rassu, A.L.; Demurtas, R.; Scarabotto, A.; et al. Exploratory screening for Fabry's disease in young adults with cerebrovascular disorders in northern Sardinia. *BMC Neurol.* **2015**, *15*, 256, doi:10.1186/s12883-015-0513-z.

S11. Lanthier, S.; Saposnik, G.; Lebovic, G.; Pope, K.; Selchen, D.; Moore, D.F.; Canadian Fabry Stroke Screening Initiative Study Group. Prevalence of Fabry disease and outcomes in young Canadian patients with cryptogenic ischemic cerebrovascular events. *Stroke* **2017**, *48*, 1766–1772, doi:10.1161/STROKEAHA.116.016083.

S12. Kinoshita, N.; Hosomi, N.; Matsushima, H.; Nakamori, M.; Yagita, Y.; Yamawaki, T.; Torii, T.; Kitamura, T.; Sueda, Y.; Shimomura, R.; et al. Screening for Fabry disease in Japanese patients with young-onset stroke by measuring α-galactosidase A and globotriaosylsphingosine. *J. Stroke Cerebrovasc. Dis.* **2018**, *27*, 3563–3569, doi:10.1016/j.jstrokecerebrovasdis.2018.08.025.

S13 Maruyama, H.; Miyata, K.; Mikame, M.; Taguchi, A.; Guili, C.; Shimura, M.; Murayama, K.; Inoue, T.; Yamamoto, S.; Sugimura, K.; et al. Effectiveness of plasma lyso-Gb3 as a biomarker for selecting high-risk patients with Fabry disease from multispecialty clinics for genetic analysis. *Genet. Med.* **2019**, *21*, 44–52, doi:10.1038/gim.2018.31.

4. SUPPLEMENTARY ACKNOWLEDGMENTS

The following physicians and centers participated in this study: Kazuhiro Aoki, Department of Cardiology, National Tokyo Hospital; Kenju Aoki, Neurological Center Agano Hospital; Nami Araya, Department of Pediatrics, Iwate Medical University; Tomoki Asai, Dialysis Center, Sagami Junkanki Clinic; Mio Ebato, Division of Cardiology, Showa University Fujigaoka Hospital; Kaoru Endo, Division of Neurology, Sendai City Hospital; Akihiro Fujii, Department of Neurology, Saiseikai Shiga Hospital; Makoto Furugen, Miyazaki Medical Association Hospital Cardiovascular Center; Nobuyuki Furuta, Division of Clinical Laboratory, Gifu University Hospital; Shoji Haruta, Department of Cardiology, Tokyo Women's Medical University, Yachiyo Medical Center; Tsuguhisa Hatano, Hatano Clinic, and Department of Cardiology Tokyo Medical University; Kyoko Hazama, Department of Neurology, Gunma Children's Medical Center; Shuma Hirashio, Department of Nephrology, Hiroshima University Hospital; Yukio Hiroi, Department of Cardiology, National Center for Global Health and Medicine; Manabu Hishida, Department of Nephrology, Nagoya University Hospital; Taro Horino, Department of Endocrinology, Metabolism and Nephrology, Kochi Medical School, Kochi University; Moritake Iguchi, Department of Cardiology, National Hospital Organization Kyoto Medical Center; Takeshi Inoue, Department of Pediatrics, Dokkyo Medical University Saitama Medical Center; Ayano Inui, Department of Pediatric Hepatology and Gastroenterology, Saiseikai Yokohamashi Tobu Hospital; Koji Ishiguro, Department of Neurology, Moteki Neurosurgery; Tetsuya Ito, Department of Pediatrics, Fujita Health University School of Medicine; Manabu Iwata, Hirosaki Stroke and Rehabilitation Center; Misako Kaido, Department of Neurology, Sakai City Medical Center; Kenji Kamiyama, Department of Neurosurgery, Nakamura Memorial Hospital; Bunta Kato, Department of Neurology, St. Marianna University School of Medicine, Yokohama City Seibu Hospital; Sanami Kawada, Department of Neurological Surgery, Okayama Kyokuto Hospital; Yuriko Kikkawa, Department of Neurology, Japanese Red Cross Society Narita Hospital; Masahiro Kikuchi, Department of Pediatrics, Hitachi General Hospital; Yukihiro Kimura, Division of Nephrology, Department of Internal Medicine, Gifu Municipal Hospital; Junya Kobayashi, Department of Vascular Neurology, National Hospital Organization Osaka Minami Medical Center; Yuji Koide, Department of Cardiovascular Medicine, Nagasaki University Hospital; Tomoyuki Kono, Department of Neurology, Kobe City Medical Center General Hospital; Toru Kubo, Department of Cardiology and Geriatrics, Kochi Medical School, Kochi University; Akiko Kumagai, Department of Internal Medicine, Division of Cardiology, Iwate Medical University; Yasuhiro Kumai, Department of Cerebrovascular Disease

and Neurology, Hakujyuji Hospital; Azumi Kumazawa, Omaezaki Municipal Hospital; Yasuhiro Maejima, Department of Cardiovascular Medicine, Tokyo Medical and Dental University; Takahiro Maruta, Neurological Center, Kanazawa-Nishi Hospital; Masaki Matsunaga, Department of Cardiology, Iwata City Hospital; Takeshi Matsushige, Department of Pediatrics, Yamaguchi University Graduate School of Medicine; Ohsuke Migita, Department of Clinical Genetics, St. Marianna University School of Medicine; Yusaku Mochizuki, Department of Cardiology, Iwata City Hospital; Takayuki Momoo, Department of Neurology, Hiratsuka Kyosai Hospital; Yasuhiro Morita, Department of Cardiology, Ogaki Municipal Hospital; Chishio Munemura, Department of Nephrology, Tottori Prefectural Central Hospital; Kumiko Muta, Department of Nephrology, Nagasaki University Hospital; Shigemi Nagayama, Department of Neurology, Kanazawa Medical University; Kentaro Nakai, Division of Nephrology and Dialysis Center, Japanese Red Cross Fukuoka Hospital; Masahiko Nakamura, Department of Cardiology, Yamanashi Prefectural Central Hospital; Yumiko Nakashima, Department of Pediatrics, Nagasaki University Hospital; Mamoru Nanasato, Department of Cardiology, Sakakibara Heart Institute; Masayasu Nishimura, Department of Nephrology, Kyoto Okamoto Memorial Hospital; Mamoru Nobuhara, Department of Cardiology, Municipal Kosai Hospital; Takahisa Noma, Department of Cardiorenal and Cerebrovascular Medicine, Faculty of Medicine, Kagawa University; Akihiko Ogata, Department of Neurology, Hokkaido Neurosurgical Memorial Hospital; Makoto Ogi, Department of Internal Medicine, Yuurinkouseikai Fuji Hospital; Kazumasa Ohara, Internal Medicine, Saiseikai Toyama Hospital; Toshiyuki Ohta, Department of Pediatric Nephrology, Hiroshima Prefectural Hospital; Yasufumi Ohtsuka, Department of Pediatrics, Faculty of Medicine, Saga University; Takayuki Okamoto, Department of Pediatrics, Hokkaido University Graduate School of Medicine; Seiji Okubo, Department of Cerebrovascular Medicine, NTT Medical Center Tokyo; Reiko Saika, Department of Neurology, Shimane University Faculty of Medicine, and Department of Neurology, National Center Hospital, National Center of Neurology and Psychiatry; Rikizo Saito, Department of Neurosurgery, Koshigaya Municipal Hospital; Katsuyuki Sakai, Sakai Medical Clinic; Akie Sakamoto, Division of Neurology and Gerontology, Department of Internal Medicine, Iwate Medical University; Shunichi Satoh, Department of Neurology, Nagano Red Cross Hospital; Hideaki Sawai, Department of Clinical Genetics, Hyogo College of Medicine Hospital; Takashi Shiihara, Department of Neurology, Gunma Children's Medical Center; Yoshiko Shimamura, Department of Endocrinology, Metabolism and Nephrology, Kochi Medical School, Kochi University; Takashi Shimozato, Department of Cardiology, Nagoya Tokushukai General Hospital; Toshiyuki Sugawara, Department of Cardiology, Aomori City Hospital; Koichiro

Sugimura, Department of Cardiology, International University of Health and Welfare, Narita Hospital; Atsushi Suzuki, Department of Cardiology, Tokyo Women's Medical University; Yoshiharu Taguchi, Fukuokamachi Taguchi Clinic; Kazue Tajima, Department of Neurology, Kameda Medical Center; Hiroki Takano, Neurology Service, Tachikawa General Hospital; Yuka Takayanagi, Division of Clinical Laboratory, Kushiro Kojinkai Memorial Hospital; Satoru Takeda, Department of Cardiology, Hiraka General Hospital; Tomoko Tamaoki, Department of Clinical Genetics, Hyogo College of Medicine; Norie Tanaka-Saito, Division of Cardiology, Kushiro Kojinkai Memorial Hospital; Takahiro Tanaka, Department of Cardiology, Showa General Hospital; Kei Tawarahara, Department of Cardiology, Hamamatsu Red Cross Hospital; Tsuyoshi Torii, Department of Neurology, National Hospital Organization Kure Medical Center; Toshinori Ueno, Department of Nephrology, Hiroshima Prefectural Hospital; Shigehisa Ura, Department of Neurology, Japanese Red Cross Asahikawa Hospital; Minoru Wakasa, Department of Cardiology, Kanazawa Medical University; Hiroyuki Watanabe, Department of Cardiovascular Medicine, Akita University Graduate School of Medicine; Masao Yamasaki, Department of Cardiovascular Medicine, NTT Medical Center Tokyo; Eiji Yamashita, Department of Cardiology, Gunma Prefectural Cardiovascular Center; Kanemitsu Yamaya, Oyokyo Kidney Research Institute; Toshiyuki Yano, Department of Cardiovascular, Renal and Metabolic Medicine, Sapporo Medical University School of Medicine; Yukako Yazawa, Department of Stroke Neurology, Kohnan Hospital; Akio Yokochi, Department of Nephrology, Kanto Rosai Hospital; Hirokazu Yokoi, Department of Cardiology, Rakuwakai Otowa Hospital; Hidetoshi Yoshitani, Department of Cardiology, Takai Hospital.
